# Update on the Complications and Management of Liver Cirrhosis

**DOI:** 10.3390/medsci13010013

**Published:** 2025-02-05

**Authors:** Hiba Fadlallah, Diala El Masri, Hisham F. Bahmad, Wassim Abou-Kheir, Jad El Masri

**Affiliations:** 1 Department of Anatomy, Cell Biology, and Physiological Sciences, American University of Beirut, Beirut 1107-2020, Lebanon; hjf05@mail.aub.edu (H.F.); jse20@mail.aub.edu (J.E.M.); 2 Faculty of Medicine, University of Balamand, Al-Kurah, Tripoli P.O. Box 100, Lebanon; diala.s.masri@std.balamand.edu.lb; 3 Department of Pathology and Laboratory Medicine, University of Miami Miller School of Medicine, Miami, FL 33136, USA; hxb592@miami.edu; 4 Faculty of Medical Sciences, Lebanese University, Beirut 1107-2020, Lebanon

**Keywords:** liver cirrhosis, chronic liver disease, complications, management, treatment

## Abstract

Liver cirrhosis represents the advanced pathological stage of chronic liver disease, characterized by the progressive destruction and regeneration of the hepatic parenchyma over years, culminating in fibrosis and disruption of the vascular architecture. As a leading global cause of morbidity and mortality, it continues to affect millions worldwide, imposing a substantial burden on healthcare systems. Alcoholic/nonalcoholic fatty liver disease and chronic viral hepatitis infection, hepatitis C (HCV) in particular, remain leading causes of cirrhosis. Despite significant advances in understanding the pathogenesis of cirrhosis, its management is still complex due to the multifaceted complications, including ascites, hepatic encephalopathy, variceal bleeding, and hepatocellular carcinoma, all of which severely compromise the patient outcomes and quality of life. This review aims at filling a critical gap by providing a comprehensive summary of the latest evidence on the complications and management of liver cirrhosis. Evidence-based therapies targeting both the etiologies and complications of cirrhosis are essential for improving outcomes. While liver transplantation is considered a definitive cure, advancements in pharmacological therapies offer promising avenues for halting and potentially reversing disease progression. This review summarizes the latest management strategies for cirrhosis and its associated complications, emphasizing the importance of early intervention and novel therapeutic options for improving outcomes and quality of life in affected individuals.

## 1. Introduction

**Figure 1:**
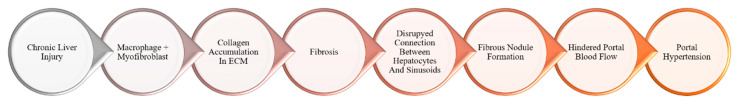
Schematic illustrating the sequence of events leading to liver cirrhosis, starting with chronic liver injury, and resulting in portal hypertension. Chronic liver injury activates macrophages and myofibroblasts, leading to collagen accumulation and fibrosis. Subsequently, a disruption in the connection between hepatocytes and sinusoids results in the formation of fibrous nodules, which hinders portal blood flow and leads to portal hypertension.

Liver cirrhosis is defined as fibrosis and the scarring of liver tissue characterized by regenerative nodules surrounded by fibrous bands as a result of chronic liver disease [1]. Chronic inflammatory liver injury leads to macrophage and myofibroblast activation in the liver, causing fibrosis secondary to collagen accumulation in the extracellular matrix. This subsequently disrupts the connection between hepatocytes and sinusoids where blood flows, leading to fibrous nodule formation and hindering portal blood flow, which ultimately leads to portal hypertension. The increased vasoconstrictor signaling and decreased vasodilator production that occur in chronic liver injury also result in restricted sinusoidal flow. Moreover, prolonged injury leads to the loss of hepatocytes and a decreased capacity for the liver to perform its normal metabolic activities [1] (Figure 1).

Cirrhosis is a significant cause of morbidity and mortality in patients with chronic liver disease [2]. In 2019, it was estimated that 2.4% of global deaths were associated with cirrhosis [2]. The worldwide prevalence of compensated cirrhosis was estimated to be 112 million cases in 2017, while that of decompensated cirrhosis was approximately 10.6 million cases [3].

Alcohol use disorder, HCV, and non-alcoholic fatty liver disease (NAFLD; also referred to as metabolic dysfunction-associated steatotic liver disease, or MASLD) account for 45%, 41%, and 26% of liver cirrhosis cases, respectively, although these causes may sometimes overlap. More recently, with the advancements in treatment and the possibility for a cure of HCV infection, the paradigm has shifted more towards NAFLD and alcohol use as the main causes of cirrhosis, with NAFLD accounting for 61.8% of incident cases and alcohol use disorder accounting for 20.0% of cases [4].

Evidence-based therapies targeting the causes and addressing the complications of liver cirrhosis have been shown to improve outcomes in these patients [5,6]. Complications of cirrhosis include ascites, hepatic encephalopathy, variceal bleeding, hepatocellular carcinoma (HCC), spontaneous bacterial peritonitis (SBP), and hepatopulmonary syndrome (HPS), among others. These complications reduce the quality of life of patients with cirrhosis [5], which is associated with symptoms such as fatigue, loss of appetite, pruritis, muscle cramps, sexual dysfunction, and poor-quality sleep [7]. Liver transplantation is currently the only curative option for cirrhosis, but there have been recent advances in pharmacological therapies aimed at halting the progression of cirrhosis and potentially reversing it [1]. This review summarizes the complications and most recent evidence regarding the management of cirrhosis.

## 2. Complications of Liver Cirrhosis

**Figure 2:**
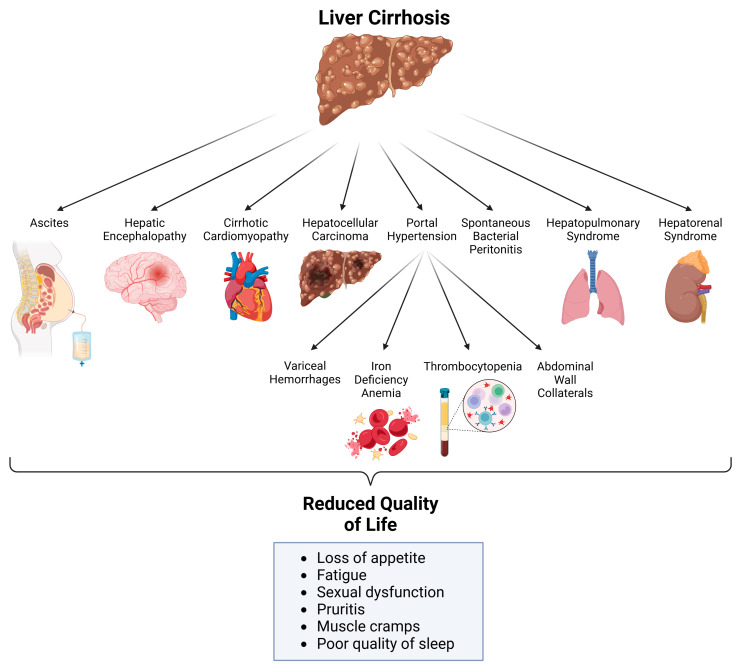
Complications of liver cirrhosis and their consequences. Created with BioRender.

Complications of liver cirrhosis include ascites, hepatic encephalopathy, cirrhotic cardiomyopathy, HCC, SBP, HPS, HRS, and portal hypertension. The latter may lead to variceal hemorrhage from different locations, iron deficiency anemia, thrombocytopenia, and the formation of abdominal wall collaterals. Subsequently, these complications cause a reduction in the quality of life in patients (Figure 2).

### 2.1. Portal Hypertension and Its Associated Complications

Portal hypertension is a critical complication of liver cirrhosis that occurs as a result of increased pressure within the portal venous system secondary to impaired blood flow due to underlying causes such as cirrhosis or portal vein thrombosis [8]. In liver cirrhosis, fibrous scarring and substantial structural changes narrow the blood vessels within the liver, increasing vascular tone in the hepatic microcirculation and resistance to blood flow in the portal vein. This raises pressure in the vein, impacting arteries and veins outside the liver. To compensate, these affected vessels widen, and new connections form. However, this attempt to increase blood flow eventually worsens the pressure in the portal vein, ultimately leading to a syndrome with increased blood flow and complications such as esophageal varices and ascites [8].

Portal hypertension is characterized by a pathological increase in the hydrostatic pressure in the portal venous area due to a rise in the pressure gradient between the portal vein and the inferior vena cava. The normal pressure gradient is less than or equal to 5 mmHg, and a pressure gradient of greater than or equal to 6 mmHg is suggestive of the presence of portal hypertension. Gastroesophageal varices and variceal bleeding may develop when the pressure gradient rises above 10 and 12 mmHg, respectively, and these complications are associated with significantly increased morbidity and mortality. Thus, a gradient greater than or equal to 10 mmHg is considered clinically significant portal hypertension [9]. Transjugular catheters are used to estimate portal pressures and determine the hepatic venous pressure gradient, which is an estimate of the pressure gradient across the liver. Vibration-controlled transient elastography and platelet counts are alternative non-invasive methods for the identification of patients with clinically significant portal hypertension [10]. Complications associated with portal hypertension include the following: variceal bleeding secondary to hemorrhage from gastroesophageal, anorectal, retroperitoneal, stomal, and other varices, thrombocytopenia due to congestive hepatopathy, the formation of abdominal wall collaterals, and acute bleeding or iron deficiency anemia due to chronic blood loss from portal hypertensive gastropathy, enteropathy, or coagulopathy.

### 2.2. Variceal Hemorrhage: Causes, Symptoms, Diagnosis, and Management

Esophageal varices are dilated submucosal distal esophageal veins that connect the portal circulation to the systemic circulation. They occur as a result of portal hypertension, increased resistance to portal blood flow, and increased portal venous blood inflow. The etiologies of portal hypertension can be divided into prehepatic, post-hepatic, and intrahepatic causes, with cirrhosis (an intrahepatic cause) accounting for most cases of portal hypertension [11]. Prehepatic causes include portal vein obstruction and massive splenomegaly with increased splenic vein blood flow. Conversely, post-hepatic causes include severe right-sided heart failure, constrictive pericarditis, and Budd Chiari syndrome with hepatic vein obstruction.

When obstruction to blood flow occurs in the portal circulation for any reason, this leads to elevated portal venous pressure. The body tries to counter this increase in pressure by developing collateral vessels. This causes portocaval anastomosis to develop in an effort to decompress the portal circulation. Considering the absence of valves in the portal venous system, increased resistance between the splanchnic vessels and the right side of the heart leads to the backflow of blood and increased pressure. The collateral vessels that develop enlarge over time, resulting in congestion in the submucosal venous plexus and tortuous dilated veins. These vessels divert blood away from the portal venous system towards the inferior and superior vena cava.

Gastroesophageal collaterals are an important component that develop secondary to portal hypertension. When these varices increase in size, they may rupture and cause severe hemorrhage. Esophageal variceal hemorrhage is the third most common cause of upper gastrointestinal bleeding, preceded only by duodenal and gastric ulcers as the first and second most common causes, respectively [11]. The pathophysiology leading to the development of esophageal varices involves increased resistance to portal blood flow due to intrahepatic vasoconstriction caused by the decreased production of nitric oxide and increased release of angiotensinogen, eicosanoids, and endothelin-1. Sinusoidal remodeling also occurs over time, which leads to disruption in the blood flow. The hyperdynamic circulation caused by splanchnic arterial vasodilation also results in increased portal flow.

Gastrointestinal bleeding episodes are commonly the first presenting symptoms among patients with varices and can be the initial presentation in patients with undiagnosed cirrhosis. Patients may present with hematemesis, hematochezia, or melena. Patients may also report a history of weight loss, anorexia, abdominal discomfort, jaundice, pruritis, altered mental status (secondary to encephalopathy), and muscle cramps. The physical exam should focus on assessing the patient’s hemodynamic stability by checking their vitals, performing an abdominal exam and palpating/percussing the liver, examining for the presence of ascites (shifting dullness), splenomegaly, visible abdominal periumbilical collateral circulation, testicular atrophy, venous hums, and anal varices.

Initial tests include a complete blood count to assess for anemia and thrombocytopenia, liver function testing (aspartate aminotransferase (AST), alanine aminotransferase (ALT), alkaline phosphatase (ALP), bilirubin, and albumin), a coagulation profile (prothrombin time (PT), partial thromboplastin time (PTT), the international normalized ratio (INR)), renal function (blood urea nitrogen (BUN) and creatinine), arterial blood gas, and hepatitis serology. In patients with variceal hemorrhage, the hemoglobin may initially be normal in active bleeding but may eventually reveal anemia. Thrombocytopenia may also be present in these patients and is the most sensitive and specific finding associated with portal hypertension and large esophageal varices [11]. AST, ALT, ALP, and bilirubin levels may be elevated, while albumin levels may be low. The BUN is also often elevated in gastrointestinal bleeds. PT and PTT may be prolonged, reflecting decreased liver function.

Imaging tests used to diagnose varices include esophagogastroduodenoscopy, which is both diagnostic and therapeutic as it can identify and treat actively bleeding varices. It can also be used to identify large varices and signs of recent bleeding and prevent rebleeding. Video capsule endoscopy screening can be used as an alternative to traditional endoscopy, and Doppler sonography is also used as a second line procedure for demonstrating the patency, diameter, and flow in the portal and splenic veins and collaterals. Other alternatives, although not routinely used, include computed tomography (CT) or magnetic resonance imaging (MRI) angiography and venous-phase celiac arteriography [12].

### 2.3. Ascites: Mechanisms, Symptoms, Diagnosis, and Treatment Options

Ascites is a common manifestation of liver cirrhosis defined by the pathological accumulation of fluid in the peritoneal cavity. Cirrhotic ascites comprise approximately 85% of cases of ascites, while the remaining 15% of cases are caused by non-hepatic etiologies [13]. The mortality associated with ascites is approximately 15% within the first year of diagnosis and 44% within 5 years of diagnosis [13]. The propagation of cirrhotic ascites is multifactorial but is generally found in the setting of portal hypertension and is mediated by aberrations in hormone and cytokine regulation and effective vascular status. Ascites typically cause symptoms such as abdominal discomfort, early satiety, nausea, vomiting, bloating, and shortness of breath. Conversely, it can also indirectly lead to other complications such as SBP and HRS.

The presence of ascites is confirmed by performing a physical exam and imaging studies. A diagnostic paracentesis is often necessary to determine the etiology of ascites and identify any possible associated infection or malignancy [14]. Laboratory tests should involve a total and differential white cell count, culture, total protein and albumin, serum-ascites albumin gradient (SAAG), cytology analysis, tuberculosis testing, lactate dehydrogenase (LDH), triglyceride, bilirubin, hemoglobin, amylase, and others if needed. The SAAG is an important measurement that can help determine if the cause of ascites is related to portal hypertension (if SAAG is >1.1 gm/dL) or not (if SAAG is <1.1 gm/dL) [15].

### 2.4. Hepatic Encephalopathy: Definition and Pathophysiology

Hepatic encephalopathy is a reversible neurologic manifestation seen in patients with advanced liver disease [16]. Hepatic encephalopathy is caused by the accumulation of neurotoxic substances in the circulation and the brain as a result of an inability of the diseased liver to clear these substances. It is characterized by neuropsychiatric abnormalities in which patients typically experience confusion, disorientation, changes in personality, and a decreased level of consciousness. In its early stages, hepatic encephalopathy manifests as a disrupted sleep–wake cycle wherein patients are awake during the night and sleep during the day. Patients subsequently experience progressively worsening levels of consciousness, ranging from confusion to lethargy, and possibly even coma and death.

Some toxins known to cause hepatic encephalopathy include ammonia (most notably), short-chain fatty acids, mercaptans, false neurotransmitters such as tyramine, octopamine, and beta-phenylethylamine (β-PEA), manganese, and gamma amino butyric acid (GABA) [17]. In healthy individuals, ammonia is normally produced by bacteria found in the gastrointestinal tract as a breakdown product of amines, amino acids, purines, and urea. It is then metabolized and cleared by the liver. However, patients with cirrhosis and advanced liver dysfunction do not have the capacity to metabolize and clear ammonia and consequently develop hyperammonemia. This may be due to a decrease in the number of functioning hepatocytes and/or portosystemic shunting. After crossing the blood–brain barrier, ammonia exerts several neurotoxic effects on the brain. Namely, ammonia alters molecular transport in astrocytes and neurons, enhances the synthesis of glutamine from glutamate by astrocytes, inhibits excitatory and inhibitory postsynaptic potential generation, impairs energy utilization due to increased GABA activity, and impairs the metabolism of amino acids [17].

### 2.5. Spontaneous Bacterial Peritonitis (SBP): Definition, Risk Factors, Clinical Presentation, and Management Strategies

Spontaneous bacterial peritonitis (SBP) is an acute infection of ascitic fluid in the abdomen that can occur in both children and adults. The source of infection is usually not identified. Most commonly, if an organism is isolated, it is identified as a Gram-negative enteric organism such as Escherichia coli or Klebsiella pneumonia [18]. These organisms account for up to 90% of isolated organisms found in SBP. This suggests that the causative agents arise from the gastrointestinal tract. Another factor frequently found in ascitic fluid is enterotoxin, which further solidifies the hypothesis that the microorganisms involved in SBP translocate from the intestinal lumen to reach the ascitic fluid.

Alternatively, it has been proposed that the contamination may be due to hematogenous spread from a distant source of infection such as a urinary tract infection. This is more likely to occur in immunocompromised patients who are already predisposed to infections. Other factors that contribute to the increased risk for infection in patients with cirrhosis include a prolonged intestinal transit time (which leads to an elevated level of bacterial overgrowth in the gastrointestinal tract), reduced protein production, decreased complement levels in both the serum and the ascitic fluid, and poor phagocytic and reticuloendothelial function. These factors all lead to a decreased capacity for the clearance of microorganisms, which further contributes to bacterial overgrowth, migration, and growth in the ascitic fluid [19].

SBP almost always occurs in patients with cirrhosis and ascites. The suspicion for diagnosis is raised when patients known to have a history of liver disease present with abdominal pain, fever, or altered mental status. However, these are not definitive criteria for diagnosing SBP as some patients may present with no abdominal pain. Fever is the most common and most useful clinical symptom in patients with SBP, as patients with cirrhosis are usually hypothermic. Thus, increased temperature in these patients is highly suggestive of SBP. Patients may also present with diarrhea, altered mental status (suggestive of encephalopathy), renal failure, or ascites that are resistant to treatment with diuretics. Expected physical exam findings include a tender abdomen, although this presentation may range from mild discomfort to guarding and rebound tenderness. SBP is typically caused by Gram-negative aerobic bacteria, with Klebsiella pneumonia accounting for up to half of SBP cases [19]. The remaining cases are caused by Gram-positive aerobic microorganisms, with *Streptococcus pneumonia* or *Streptococcus viridans* as the most common [20].

The ascitic fluid in SBP typically has a high oxygen tension, which explains why anerobic organisms are rarely seen. Most cases of SBP are caused by a single infecting organism, but there have been some cases that involved polymicrobial infection. These organisms typically arise from the gastrointestinal tract, from where they translocate to mesenteric lymph nodes and subsequently invade the ascitic fluid. Patients with decompensated liver cirrhosis are at the highest risk of developing SBP. Previous history of SBP, decreased complement levels and hepatic protein synthesis, prolonged PT, low ascitic protein levels (<1 g/dL), and prolonged proton pump inhibitor therapy (leading to increased gastric pH, thus promoting bacterial growth and translocation) are additional risk factors for the development of SBP.

### 2.6. Hepatorenal Syndrome (HRS): Pathophysiology, Clinical Features, Diagnostic Criteria, and Therapeutic Approaches

The term “hepatorenal syndrome” was first established in 1939 to describe renal failure that occurs following biliary surgery or hepatic trauma [21]. The term was later used to describe other types of acute renal failure occurring in the setting of liver disease. The International Club of Ascites (ICA) later suggested a new definition and diagnostic criteria for HRS in 1996, which have since been acknowledged and used to describe functional renal failure that occurs in patients with advanced cirrhosis [22,23]. These criteria have since been updated. Further research suggested that renal failure secondary to cirrhosis was functional. This was confirmed by the occurrence of HRS despite normal kidney histology and no evidence of proteinuria. Clinically, this was confirmed by the improvement of kidney function seen in patients with liver cirrhosis who underwent a liver transplant [24].

In developing countries, viral hepatitis is the most common cause of liver failure (and subsequent HRS). Conversely, in developed countries, the most common causes are medications (particularly cytochrome p450 inducers), alcoholism, and non-alcoholic steatohepatitis. Less commonly, liver failure leading to HRS can be caused by viruses such as cytomegalovirus (CMV), human herpesvirus 6 (HHV6), and parvovirus B19. Finally, vascular pathologies such as hepatic/portal vein thrombosis can also lead to liver failure and HRS. HRS occurs in approximately 4% of patients with decompensated liver disease [24]. Of these, most patients have portal hypertension secondary to alcoholic hepatitis, cirrhosis, or metastatic cancers.

The pathophysiology of HRS is not completely understood but is believed to involve a complex neurohormonal cascade triggered by cirrhosis and portal hypertension. This cascade induces the production and release of cytokines and vasodilators, such as nitric oxide and prostaglandins, leading to splanchnic and systemic vasodilation. The resultant drop in systemic blood pressure activates carotid and aortic baroreceptors, which initiates compensatory mechanisms, including activation of the renin–angiotensin–aldosterone system, the sympathetic nervous system, and the release of vasopressin. As cirrhosis progresses further, it leads to a fall in cardiac output and a subsequent fall in systemic vascular resistance. This creates a vicious cycle in which the fall in systemic pressures induces further renal vasoconstriction, causing further renal hypoperfusion, which is worsened by renal vasoconstriction. The end result is renal failure. HRS is commonly precipitated by SPB, the large volume paracentesis of ascitic fluid without plasma expansion, and gastrointestinal bleeding [25].

HRS is a diagnosis of exclusion and was historically classified into two types. Type 1, which is characterized by a rapid and progressive decline in renal function represented by doubling of the serum creatinine to at least >2.5 mg/dL or a decrease in creatinine clearance by greater than or equal to 50% over a two-week period [26], a urine output of <500 mL/day, low urinary sodium excretion, and a normal urine sediment. Conversely, type 2 HRS is characterized by less severe kidney injury and a progressive slower course of moderate renal failure with serum creatinine concentrations between 1.5 and 2.5 mg/dL and diuretic resistant ascites. This definition has been revised by the ICA, and the older classifications, the 2-week time limit needed to diagnose type 1 HRS, and the 2.5 mg/dL threshold for serum creatinine were removed. According to the new definition, HRS is now divided into HRS-AKI (formerly called type 1 HRS) and HRS-non-AKI (formerly called type 2 HRS). HRS-AKI is defined as worsening renal function in patients with advanced cirrhosis that meets the ICA-AKI criteria, does not respond to volume expansion with albumin, has had no recent exposure to nephrotoxic agents, and no evidence of shock or signs of structural kidney disease, as defined by proteinuria (<500 mg/dL), hematuria (<50 red blood cells per high power field), and normal renal ultrasonographic findings [27]. The new definition depicts a new phenotypic classification of HRS-AKI in patients with cirrhosis based on pathophysiological characteristics. Conversely, HRS-non-AKI is now used to describe other causes of AKI that occur in patients with cirrhosis (besides HRS-AKI). These causes include bile salt nephropathy, prerenal hypovolemia secondary to bleeding, excessive diuretic loss or any major fluid loss, acute tubular injury, acute tubular necrosis, and interstitial nephritis [27]. It is important to note that other causes of acute kidney injury should be excluded before diagnosing patients with HRS.

### 2.7. Hepatocellular Carcinoma (HCC): Pathophysiology, Epidemiology, and Risk Factors

HCC is one major cause of death among cirrhotic patients, accounting for more than 50% of deaths [28]. Although the rates of mortality from cirrhosis have been trending down, those from HCC were increasing. Approximately 80 to 90% of cases of HCC were found on autopsies to have cirrhosis [29]. HCV infections were identified in the most number of cases, followed by hepatitis B (HBV), alcohol use, and hemochromatosis then other less common causes [30]. In patients with cirrhosis, HCC is more prevalent in males than females, and more in older patients than young ones.

Many risk factors for HCC have been identified so far in cirrhotic patients. These include male gender, age, obesity, years with cirrhosis, family history of liver cancer, baseline alpha-fetoprotein (AFP), albumin, and AST [31]. Using the ADRESS-HCC risk model, the l-year probability of HCC in various liver diseases was examined on patients. This model found age, gender, race, diabetes mellitus, the etiology of cirrhosis, and its severity to be independently associated with HCC [32].

Cirrhosis, characterized by chronic liver disease with fibrosis and inflammation, causes histological abnormalities in the liver, which are recognized as a significant risk factor for hepatocellular carcinoma. In cirrhosis, viral infections, chronic necroinflammation, and the generation of reactive oxygen species can impair the genetic integrity of proliferating hepatocytes, resulting in mutations that may drive cancer development [33]. Senescence results from necroinflammation, the deposition of extracellular matrix, and the shortening of telomeres [34]. Several studies confirmed a role for each of NF-κB, STAT3 and JNK in linking cirrhosis to HCC, in addition to inflammasomes, which were considered contributors in this field [35,36].

### 2.8. Hepatopulmonary Syndrome (HPS) and Portopulmonary Hypertension (PPH)

Hepatopulmonary syndrome (HPS) and portopulmonary hypertension (PPH) are pulmonary vascular complications that occur as a result of advanced liver disease. However, these syndromes are distinct in their pathophysiology, and the clinical implications and management of these syndromes also vary.

HPS is characterized by impaired arterial oxygenation resulting from intrapulmonary vascular dilations in patients with advanced liver disease. While most commonly associated with cirrhosis and portal hypertension, HPS has also been reported in acute liver failure, non-cirrhotic portal hypertension, congenital portosystemic shunts, and chronic hepatitis. Characteristic histological features include the gross dilation of pulmonary precapillary and capillary vessels reaching a diameter of 100 microns as well as an increase in the total number of dilated vessels [37]. Portal venule thrombosis and widespread vascular proliferation were also observed in explanted livers of patients with HPSS who underwent liver transplantation. These findings suggest that the changes that occur in the liver vascular bed may contribute to the development of HPS [37].

Some factors that may predispose to the development of HPS include the presence of hepatic dysfunction and a decrease in venous blood flow in the liver [38]. Imaging in patients with cirrhosis and HPS revealed an increased obstruction of intrahepatic portal branches, hepatojugular blood flow, and relatively more portosystemic shunts than patients with cirrhosis but no HPS. Four main pulmonary changes have bene implicated in the pathogenesis of HPS, namely; (1) reduced vascular tone via increased nitric oxide levels and endothelial dysfunction induced by endothelin-1, (2) pulmonary vascular infiltration by monocytes, likely resulting from the increase in bacterial translocation from the gut, (3) vascular remodeling and angiogenesis leading to intrapulmonary arteriovenous shunt formation, and (4) diminished ventilation and diffusion capacity secondary to dysfunctional alveolar type II cells [38].

In HPS, abnormal oxygenation is due to three separate mechanisms. The first and most important is the dilation of the pulmonary capillaries, which enables the rapid passage of mixed venous blood into the pulmonary veins. This ultimately leads to a ventilation–perfusion mismatch. The second mechanism by which abnormal oxygenation occurs is via direct right-to-left shunting that occurs secondary to the formation of pleural and pulmonary arteriovenous connections. Finally, the increase in the diffusion distance resulting from dilated or thickened capillaries also leads to a decrease in the diffusion capacity [38].

Patients with HPS often present with dyspnea, although this is nonspecific as patients with cirrhosis may have dyspnea due to other causes such as ascites, hepatic hydrothorax, PoPH, anemia, and decreased functional status. Similarly, other physical exam findings such as spider angiomas, cyanosis, and clubbing may be present in patients with HPS but are nonspecific findings. Findings more specific for HPS include platypnea and orthodeoxia, defined as increased dyspnea when sitting up from a supine position and a drop in oxygen saturation of greater than or equal to 5% when moving from a supine to an upright position, respectively. Nevertheless, these findings are not pathognomonic, and HPS should be suspected in all patients with chronic liver disease. The triad of hepatic dysfunction, impaired arterial oxygenation, and intrapulmonary vasodilation is characteristic of HPS.

To diagnose HPS, patients should fit the following criteria: (1) chronic liver disease, non-cirrhotic hypertension, or other causes of cavopulmonary shunt; (2) arterial hypoxemia on an upright, room air arterial blood gas defined as an alveolar–arterial (A-a) oxygen gradient ≥ 15 mmHg or ≥20 mmHg if ≥65 years old; and (3) a positive contrast-enhanced transthoracic echocardiogram showing intrapulmonary vascular dilations [38,39].

PPH is another complication of cirrhosis that remains underdiagnosed. PPH is defined as pulmonary arterial hypertension (with a mean pulmonary artery pressure greater than 25 mmHg and pulmonary capillary wedge pressure less than 15 mmHg) in a patient with coexisting portal hypertension with or without intrinsic liver disease and no findings suggestive of an alternative cause of PAH. The pulmonary vascular remodeling seen in PPH is pathologically similar to that seen in idiopathic PAH on autopsy and lung explant studies [40]. Findings observed in PPH include the vasoconstriction of pulmonary arteries, medial hypertrophy secondary to smooth muscle proliferation, and intimal fibrosis that eventually leads to plexogenic arteriopathy. These alterations correspond to platelet aggregation and in situ thromboses, which cause pulmonary artery obstruction. The mechanisms underlying these changes are not yet understood, but hepatic injury, splanchnic vasodilation, hyperdynamic state, and portosystemic shunting are suggested mechanisms that may increase the production and decrease the clearance of circulating factors such as endothelin-1, interleukin-6, thromboxane A2, serotonin, vasoactive intestinal peptide, estrogen, and other interleukins that enable remodeling [41]. Notably, the levels of these circulating factors were increased in patients with cirrhosis and PPH, while the levels of prostacyclin were decreased when compared to patients with cirrhosis but no PPH.

Patients with PPH commonly present with a chief complaint of exertional dyspnea. However, they may also have dyspnea at rest as well as other non-specific symptoms such as chest pain, palpitations, presyncope, and syncope. On physical exam, patients have signs of pulmonary hypertension and right heart failure. These include an accentuated and split S2, right ventricular heave, right sided S3 gallop, jugular venous distention, ascites, and lower extremity edema. However, some patients with mild PPH may be asymptomatic. Patients may also have mild hypoxemia, but severe hypoxemia with clubbing or cyanosis is rare. To diagnose PPH, patients should have a clinical diagnosis of portal hypertension and evidence of pulmonary arterial hypertension defined as follows: (1) mean pulmonary artery pressure (mPAP) > 25 mmHg on right heart catheterization (RHC); (2) peripheral vascular resistance > 3 Woods units (240 dynes/s per cm^−5^); and (3) pulmonary capillary wedge pressure < 15 mmHg. The severity is graded as mild (25 ≤ mPAP < 35 mmHg), moderate (35 ≤ mPAP < 45 mmHg), and severe (mPAP ≥ 45 mmHg) based on RHC findings [38].

### 2.9. Cirrhotic Cardiomyopathy

In cirrhosis, there is a combination of autonomic dysfunction, alterations in cell membrane composition, ion channel defects and an overproduction of cardio-depressant factors. This leads to hypovolemia, prolonged QT, cardiomyocyte apoptosis, and eventually eccentric left ventricular hypertrophy and diastolic dysfunction. Due to poor energy metabolism, systolic activity can also be compromised [42]. Histologically, there might be fibrosis, subendocardial edema, and vacuolation of the nucleus and cytoplasm of myocardial cells [43].

The management of cirrhotic cardiomyopathy is generally similar to that of heart failure, including angiotensin-converting enzyme (ACE) inhibitors, angiotensin receptor blockers (ARBs), loop and thiazide diuretics, aldosterone receptor antagonists, and beta-blockers. However, as to all other complications of cirrhosis, liver transplant remains the best solution [44].

## 3. Management Strategies for Liver Cirrhosis

### 3.1. General Management Principles

The main target of the management of cirrhosis is to prevent complications, liver decompensation, and death. These targets require frequent prevention counseling, monitoring, and management by specialized physicians.

#### 3.1.1. Addressing the Underlying Cause of Cirrhosis

The first step in managing cirrhosis is based on treating the specific etiology and is best started at the earliest time possible. Below, presented are the most important causes of liver cirrhosis, with their first line treatment, all classified based on their etiology [45]:

- Viral hepatitis B, C, and D: anti-viral treatment;
- Autoimmune hepatitis: prednisone and azathioprine;
- Cholestatic-primary biliary cholangitis and primary sclerosing cholangitis: ursodeoxycholic acid for biliary cholangitis, yet no proven therapy for sclerosing cholangitis;
- Metabolic hemochromatosis, NASH, alcohol-related liver disease, Wilson disease, alpha-1 antitrypsin deficiency: iron depletion, weight loss through nutritionist referral, medical therapies, and bariatric surgery, alcohol abstinence, penicillamine, and augmentation therapy, respectively.

#### 3.1.2. Lifestyle Recommendations and Dietary Modifications

##### Dietary Aspect

- Malnutrition in Cirrhosis

Liver metabolic balance is disrupted in cirrhotic patients, leading to an increased protein catabolism, decreased glycogen synthesis, and increased lipolysis. This malnutrition is usually caused by reduced nutrient intake, impaired intestinal absorption, increased protein loss, disrupted metabolism and increased inflammation [46].

Sarcopenia, which refers to protein-energy malnutrition due to a reduction in muscle mass or function, is a common complication of cirrhosis, with an estimated rate of 20 to 60%, leading to an increase in disease severity [47]. It is a key objective feature of malnutrition that can coexist with obesity in approximately 35% of cases awaiting liver transplantation, increasing the rates of morbidity and mortality [48].

Sarcopenia is associated with several adverse outcomes, such as functional limitations, impaired quality of life, infections, hepatic encephalopathy (HE), and increased mortality, primarily due to cardiopulmonary muscle impairment [49]. In the clinical setting, sarcopenia is assessed using mid-arm muscle circumference measurement, CT scan imaging (considered the most accurate), quadriceps ultrasound imaging, or physical frailty evaluation [47,50].

As for its management, it involves a focus on lifestyle, nutrition, and exercise [51]. Alcohol and smoking cessation, improving sleep quality, and enhancing psychological health are essential, along with dietary management. Current clinical practice guidelines recommend a caloric energy and protein intake of 25–35 kcal/kg/day and a protein intake of 1.2–1.5 g/kg/day in compensated cirrhosis (Child–Pugh A) and 30–35 kcal/kg/day and 2.0 g/kg/day, respectively, in decompensated cirrhosis (Child–Pugh B–C) [52]. Additionally, exercise improves cardiovascular fitness and muscle mass, with moderate-intensity routines being ideal. These should include aerobic exercises (3 days/week) and resistance training (2 days/week), performed at a moderate intensity [50].

- Obesity in Cirrhosis

Obesity is considered to have a major role in the prognosis of cirrhosis. Its frequency in compensated cirrhosis cases ranges from 20 to 40%, comparable to that in the general population [53]. In patients with compensated cirrhosis, obesity increases the risk of clinical decompensation, portal vein thrombosis, portal hypertension, and HCC [53]. As for decompensated cirrhosis, obesity is associated with an increase in the risk of bacterial infections resulting in admission to the intensive care unit (ICU) [54]. Even after liver transplantation, obesity increases the probability of severe complications and is sometimes considered a contraindication to the surgery in the first place [55].

The reduction in body weight has been shown to improve outcomes of compensated cirrhosis. The loss of 10% of an obese patient’s weight yields the best results, suggesting that weight loss could become a lifestyle intervention. However, since weight loss can cause adverse effects such as sarcopenia, ascites, and HE, special care is required in decompensated cases of cirrhosis [56].

Therefore, a dietitian/nutritionist with expertise in the management of liver diseases is needed. Another treatment option for obese patients with compensated disease is bariatric surgery, yet lifestyle changes remain the cornerstone of treatment in cirrhosis [57].

##### Detrimental Lifestyle Factors

- Physical Activity and Exercise

Exercise interventions provide significant benefits for sarcopenia, cardiorespiratory fitness, insulin sensitivity, and quality of life. The objectives of exercise interventions are achieved by increasing muscle mass, enhancing muscle function, and preventing additional breakdown [58]. They are considered safe in cirrhotic patients, even those at the decompensated stage [59]. Recommendations for exercise programs are to combine both aerobic and resistance training, with a moderate intensity for a minimum duration of 8 to 12 weeks [60].

- Alcohol

In cirrhosis, even a moderate alcohol intake can worsen the prognosis by increasing the portal pressure [61]. However, abstinence causes the histological regression of cirrhosis, the improvement of portal hypertension, and reductions in the risk of clinical decompensation [62]. The risk of having HCC has also decreased, which leads, together with previously mentioned effects, to improved survival rates following over a 20% drop in deaths at 5 years [63]. A synergistic effect between alcohol and obesity is found on liver damage, leading to a higher risk of cirrhosis and HCC [64].

- Cigarette Smoking

Cigarette smoking significantly increases the risk of liver cirrhosis and is found to stimulate fibrogenesis [65]. Also, smoking increases the incidence of HCC, leading to a worse survival, especially in cirrhotic patients [66]. As for liver transplantation cases, smoking increases mortality due to non-graft related causes, mainly due to increasing cardiovascular events and cancer onset [67]. Based on this, it may be beneficial to add smoking cessation to the list of lifestyle adaptations in all patients with cirrhosis.

- Poor Oral Health

Cirrhotic patients have poor oral health with high rates of periodontitis, independent from the different etiologies. Several studies linked periodontitis to an additional rate of death in cirrhosis, by linking oral health to the gut–liver axis [68]. Therefore, the care for patients’ oral health is an important step in managing the lifestyle of cirrhotic patients.

#### 3.1.3. Nutritional Support and Vitamin Supplementation

##### Mediterranean Diet

It has been found that a diet rich in vegetables, fruits, and olive oil reduces the risk of complications in cirrhosis, possibly by acting on gut microbiota. By decreasing insulin resistance, this diet also reduces some complications of cirrhosis, mainly those involving the cardiovascular system, in addition to decreasing the risk of HCC [69]. This makes a Mediterranean diet of great value in cirrhotic patients.

##### Vitamin Supply

Vitamin deficiency, especially the lipid-soluble vitamins, is usually present in advanced liver diseases due to hepatic disorders or diminished reserves. The majority of patients requiring liver transplantation had vitamin deficiency, namely, vitamin A and D deficiencies [70].

- Vitamin A

Hepatic stellate cells are the main storage site for vitamin A. Several studies have reported vitamin A deficiency in cirrhosis, mainly in hepatitis C- and alcohol-related cirrhosis [71]. Caution must be exercised when correcting this deficiency, since high doses can cause hepatotoxicity.

- Vitamin D

The liver is the site for vitamin D 25-hydroxylation, and its deficiency in cirrhosis. Similar to vitamin A deficiency, it is primarily linked to hepatitis C- and alcohol-related cirrhosis [72]. Restoring normal levels of this vitamin is crucial, as its deficiency has been associated with increased mortality rates [73].

- Vitamin E

Vitamin E deficiency is also prevalent in cirrhosis, and, depending on the specific etiology, its supplementation has been found to benefit liver diseases. This effect has been particularly well documented in alcoholic liver disease [74]. Supplementation has been shown to result in significant improvement in terms of liver enzymes and markers of steatosis and inflammation [75].

#### 3.1.4. Regular Monitoring and Specific Treatments for Complications

Every six months, laboratory tests such as basic metabolic panel, liver function tests, complete blood count, and PT/INR should be conducted [76]. These labs aid in calculating the Child–Pugh and Model for End-Stage Liver Disease (MELD) scores, which are used to assess the prognosis of cirrhosis [77].

As for the complications of liver cirrhosis, some have common screening and management methods, and others have unique procedures. Table 1 summarizes the complications, their probability, screening, and treating methods.

#### 3.1.5. Screening Guidelines for HCC

As for HCC, international professional society guidelines recommended screening for HCC in cirrhotic patients, despite the various etiologies [90,91]. Screening is limited to cases where patients benefit from detecting HCC early, namely, those with compensated cirrhosis (Child–Pugh class A or B) [92]. Screening is not required for patients with a life expectancy of 12 months or less, in addition to those of class C Child-Pugh, except for the cases listed for liver transplant.

Imaging is mainly the technique of screening; however, the American guidelines leave it to the clinician to test for AFP as a biomarker for HCC [91,93]. It is recommended by most guidelines to screen every 6 months, as it was found that increasing the frequency to every 3 months did not improve detection rates yet increased the detection of nonspecific nodules [94].

Despite being operator-dependent and lacking the exploration of some liver areas, ultrasonography is considered the imaging modality for HCC screening, due to its low cost, noninvasive procedure, and accuracy [90,95]. However, its sensitivity for detecting early-stage HCC in cirrhotic patients was found to be about 45% [96]. In cases where ultrasound is inadequate, such as in multinodular cirrhosis, other alternatives can be used, namely, MRI and CT [97].

### 3.2. Management of the Complications of Cirrhosis

#### 3.2.1. Prevention and Management of Variceal Bleeding in Cirrhotic Patients

In patients with medium to large variceal bleeding, primary prophylaxis must be considered. Three preventive measures are used in practice, including traditional non-selective beta blockers (NSBBs), mainly propranolol and nadolol, beta blockers with alpha adrenergic blocking effects, namely, carvedilol, or endoscopic variceal ligation (EVL).

##### NSBBs

NSBBs are anti-hypertensive medications used in cirrhosis for the reduction in portal hypertension, specifically the hepatic venous pressure gradient (HVPG). Beta blockers, propranolol most commonly, act on beta-1 cardiac receptors, inducing a negative chronotropic effect and decreasing the cardiac output [98]. They also act on the splanchnic beta-2 receptors. This decreases portal blood flow by constricting the splanchnic arterial bed [99]. In general, a reduction of 20% or more in the HVPG is the goal of NSBB use [100].

The two main NSBBs used are propranolol and nadolol, each given at gradually increased doses till reaching a heart rate no less than 55 to 60 bpm. Propranolol is started at 20 to 40 mg twice per day, and the dose increases gradually to reach a maximum of 320 mg per day in patients who do not have ascites and 160 mg per day for those who have ascites. Nadolol is started at the same dose of 20 to 40 mg but once per day, reaching a maximum of 160 mg per day in patients without ascites and 80 mg per day in patients with ascites [101].

NSBBs are indicated in cases of high risk of rupture, identified by red wale marks on the surface of the varices, and classified as Child–Pugh C [102]. Some studies have shown that the use of NSBBs in patients with end-stage cirrhosis might be less effective, and it might increase the risk of developing HRS. Mortality would also increase due to the negative effect on the cardiac compensatory reserve [103]. However, other studies show no similar effects, with no negative impact [104]. The use of NSBBs is contraindicated in patients with a low systolic blood pressure (<90 mmHg), acute kidney injury, or serum sodium < 130 mEq/L [101]. It must also be stopped in cases of SBP, sepsis or bleeding, and EVL is considered in case NSBBs cannot be continued again in 3 to 6 days [93].

##### Carvedilol

Carvedilol is an NSBB that acts also on alpha-1 cardiac receptors, blocking these receptors and therefore promoting an additional sinusoidal vasodilation. This leads to a larger reduction in the HVPG than that seen with NSBBs, but the superiority of carvedilol over NSBBs is still under studies [105]. Carvedilol is started at a dose of 6.25 mg per day, reaching 12.5 mg per day after three days unless the systolic blood pressure decreases to less than 90 mmHg [101].

##### EVL

EVL is the placement of a rubber ring or band on the variceal columns, resulting in occlusion from thrombosis. This leads to necrosis and sloughing off of the variceal tissue, leaving a healing-capable ulcer. EVL must be performed every 2 to 8 weeks until the varices are completely eradicated, then follow-ups with endoscopy must be performed at a lower frequency [101].

#### 3.2.2. Management of Ascites in Cirrhotic Patients

##### Diuretic Therapy

One common complication of liver cirrhosis is ascites in the abdomen. Some studies infer some benefit seen with sodium restriction [106]; however, diuretics are the first-line therapy so far. The most common diuretic in use is spironolactone, sometimes aided by furosemide or bumetanide, whereas amiloride is a less used alternative.

##### Spironolactone

Spironolactone is the first choice for the treatment of ascites in a cirrhotic patient, as it is a potassium-sparing aldosterone antagonist that increases natriuresis at the distal tubules [107]. Doses are administered and are increased at a gradual pace (Table 2). Spironolactone induces a delayed natriuretic effect 3 to 5 days post-administration [108]. However, spironolactone inhibits androgenic activity leading to adverse effects including reduced libido, gynecomastia in men, or disturbed menstruation in women [109]. One other side effect to be monitored closely is hyperkalemia [110].

##### Furosemide

For ameliorated natriuretic effects, the loop diuretic Furosemide is mainly used with spironolactone rather than alone [111]. The dosages are also increased gradually and cautiously every 2 to 3 days not to induce electrolyte disturbances or metabolic alkalosis [112].

##### Other Diuretics

Amiloride is an alternative but less effective diuretic that acts on the distal tubule and is sometimes used for the treatment of ascites in cirrhotic patients [113]. In general, diuretics must be started after the failure of dietary salt restriction, and furosemide is only added to spironolactone if the maximal dosage does not produce the desirable effect [114].

##### Paracentesis

For patients with large or recurring ascites, total paracentesis has been proved effective with all ascetic fluid drained till dry within 1 to 4 h. As a compensator volume expander, 8 g of albumin must be infused for every liter of ascetic fluid removed to ensure reduced complications including possible circulatory dysfunction or renal function and electrolyte disturbances [79,115]. Reinitiating diuretic therapy after paracentesis has been shown to markedly decrease recurrence of ascites [116].

#### 3.2.3. Prophylaxis and Management of SBP

##### Antibiotic Prophylaxis for SBP

No prophylaxis is required in patients with no history of SBP and ascitic fluid protein less than 10 g/L [117]. However, those who had previous episodes of SBP must be started on oral norfloxacin at 400 mg/day, therefore reducing the probability of recurrence as well as SBP due to Gram-negative bacilli [118]. Ciprofloxacin is also being used for prophylaxis [119].

##### Treatment of SBP: Empirical Antibiotics

Once a diagnosis of SBP is made, empiric treatment must be directly started to reduce complications and mortality. Table 3 summarizes the antibiotics and the cases each is used for [120]. Higher scores on the Quick Sequential Organ Failure Assessment (SOFA) and Acute Physiology and Chronic Health Evaluation (APACHE II) are indications for more aggressive treatment [121]. In general, antibiotics are given for 5 to 7 days [122].

##### Treatment of SBP: The Use of Albumin

The use of albumin was shown to decrease the risks for HRS and death associated with SBP [123].

##### Treatment of SBP: Other Recommendations

Diuretics and NSBBs are contraindicated in patients with SBP, and they must be discontinued [27,124].

#### 3.2.4. Management of HRS in Cirrhotic Patients

For the treatment of HRS, the causal factors must be identified and removed, including the discontinuation of diuretics and NSBBs if present [125,126]. Albumin is used at this point for its anti-inflammatory and antioxidant effects, as well as its ability to clear various bacterial products [127,128]. Vasoconstrictors are used along with albumin to improve the hemodynamic state leading to HRS [129]. Table 4 summarizes the main vasoconstrictors and their effects [130]. Renal replacement therapy, such as hemodialysis or kidney transplantation, has been shown in some studies to have beneficial effects in patients who will receive liver transplants within few weeks to months [131] and who have not improved with pharmacological therapy.

#### 3.2.5. Management of Hepatocellular Carcinoma in Cirrhotic Patients

Three possible treatment options are available for HCC in cirrhotic patients. Surgical resection is most efficient for solitary tumors with preserved liver functions and no significant portal hypertension [132]. Recurrence rates of 70% were identified at 5 years when surgical resection was performed. However, liver transplantation is considered an optimal intervention as it also treats the main cause, which is cirrhosis, but choosing transplantation is limited by the lack of donors [133]. A potential alternative to both surgical resection and liver transplant is local ablation, either using radiofrequency or percutaneous ethanol injections. This results in injury and necrosis of the tumor cells [134].

#### 3.2.6. Management of Hepatic Encephalopathy in Cirrhotic Patients

As for the management for hepatic encephalopathy, non-absorbable disaccharides and antibiotics are the main treatment options. Non-absorbable disaccharides, mainly lactulose and lactitol, reduce the colonic pH, therefore reducing colonic bacteria and ammonia production [135]. The acidic environment also aids in converting ammonia to ammonium. Similarly, non-absorbable antibiotics, namely, rifaximin, decrease gut bacteria, thereby decreasing the production of ammonia [136]. New treatment options are still under investigation, including some amino acids, laxatives and antimicrobials, but clinical trials are still incomplete [137].

### 3.3. Liver Transplantation

Liver transplantation (LT) is a lifesaving intervention for the management of advanced liver diseases that restores health and extends lifespans [138]. Liver cirrhosis is the most common cause for liver transplantation worldwide, where around 80% of cases are due to decompensated cirrhosis [139]. The Scientific Registry of Transplant Recipients declared that, following liver transplantation, the rate of patient survival is 90% at 1 year and is 77% at 5 years, following deceased donor liver transplantation [140].

#### 3.3.1. Indications for Liver Transplantation in Cirrhotic Patients

In general, LT is not indicated in all cirrhotic patients, especially since the scarcity of donor organs necessitates the prioritization of less healthy cases [141]. As a gray zone between total requirement and non-indicated cases, LT is only appropriate in some cirrhosis cases, which are as follows: (1) decompensated cirrhosis, such as ascites, variceal hemorrhage, HE, and jaundice, occurring at a rate of 5–7% per year and reducing median survival more than 10 years [142], or (2) the Model of End-Stage Liver Disease score (MELD) ≥ 15, which was correlated to a higher mortality in the first year on the waiting list compared to those receiving a liver [143].

There are several contraindications for LT, some of which are absolute, such as severe cardiopulmonary disease, uncontrolled sepsis, active extrahepatic malignancy, brain death, anatomic or technical barriers, and active alcohol or illicit substance abuse [141]. Other contraindications are relative, which are mainly institution and patient-dependent [141].

#### 3.3.2. Evaluation and Selection of Candidates for Liver Transplantation

The evaluation for being listed on the waiting list for liver transplantation is usually performed by a committee assessing the physical, mental, and social health of the patient. In general, to be a priority for receiving a liver, the patient must be sick, yet healthy enough to undergo the surgery and recover from it [144].

As for liver allocation, patients are classified into status 1 patients, who have the highest priority of receiving an organ, and other patients that are classified based on the MELD (>12 years of age) or Pediatric End-Stage Liver Disease (PELD) (<12 years of age). Based on UNOS regulation, the priority in organ allocation is to local status 1 candidates, followed by regional ones if no locals are available [145].

Status 1 patients divides into groups A and B, the first including patients >18 years old having fulminant liver failure, the primary non-function of a transplanted liver, or hepatic artery thrombosis and the second including patients <18 years having chronic liver disease and hospitalized to the intensive care unit with a MELD or PELD score > 25, in addition to being on mechanical ventilation, having gastrointestinal bleeding that requires 30 mL/kg of red cell replacement, having renal insufficiency requiring dialysis, or having a GlasgowComa Score < 10 [146].

The MELD is a validated scoring tool used to estimate the 90-day mortality risk in liver transplant candidates, based on creatinine, bilirubin, and INR levels, assessing the disease severity and predicting short-term mortality post-transplantation. Lately, other factors (albumin and gender) were added to this tool, leading to a newer assessment model (MELD 3). It was shown more accuracy in terms of mortality prediction [147]. Similarly, PELD is a predicting score based on albumin, bilirubin, INR and growth component, in addition to a specific consideration for ages below 1 year [147].

Since its introduction of the MELD, it was noticed that some medical conditions were underestimated by this score in terms of severity and mortality, especially since it focuses on short-term mortality. Therefore, exception categories were added, balancing the mortality risk and the access to liver transplantation on the waiting list [148]. These exceptions include patients having HCC, hepatopulmonary syndrome, and hereditary ATTR-amyloidosis, among others [149].

Recently, another scale named the Liver Frailty Index was introduced to assess frailty in patients with cirrhosis. This has provided an objective functional measure independent of age that was found to be appropriate at capturing mortality risk, which has been useful in clinical transplant practice in the outpatient setting [150].

#### 3.3.3. Surgical Procedure and Post-Transplant Care

##### Standard Surgical Technique

Liver transplantation is a surgical procedure involving the donor and the recipient at the same time. Starting with a total hepatectomy of the native liver in the recipient, the ligaments attaching to the liver are divided, followed by the dissection of the bile duct, hepatic artery, and portal vein (hilar structures). These steps prepare the recipient for the implantation of the donor’s liver. Also, the inferior vena cava (IVC) is encircled to assure vascular control. Vascular clamps are inserted on the portal vein, followed by the removal of the liver. Meanwhile, the donor’s liver is surgically prepared for transfer to the operative field [151]. Several centers differ between living and deceased liver donors, with a previous superiority to the latter. However, living donation has grown recently, and several studies reached comparable outcomes to dead-donation transplantations [152].

##### Procedure for Deceased-Liver Transplantation

In cases of deceased donors, the whole liver is more commonly transplanted. In order, the supra-hepatic IVC is connected, followed by the infra hepatic IVC and the portal vein. Then, the clamps are removed, allowing blood to perfuse the liver tissue. Next, hepatic arteries are connected at a location near the gastroduodenal artery junction in around 80% of the cases, and the bile duct is reconstructed [151]. 

Recently, split liver transplantation, a surgical technique with favorable outcomes, is being more studied, with the aim to increase the number of recipients and maximize the benefit from each donation. This classical form of this surgery divides the liver into a left lateral lobe graft (segments II + III), which is transplanted in a pediatric or small adult recipient, and an extended right lobe graft (segments I + IV–VIII), which is transplanted for an adult recipient [153]. However, despite some studies reporting comparable outcomes, other studies found a higher risk of complications following this type of surgery using deceased-donor liver compared to whole liver transplantation [154].

##### Procedure for Living-Liver Transplantation

Unlike deceased donation, living donor graft only includes a part of the liver, where the liver regenerates in both the donor and recipient [155]. Different grafts can be taken from the donor, depending on the specific and its best fit, including the following: (1) left lateral sector, comprising around 20% of the total liver volume, (2) the left lobe, compromising around 40% of the total liver volume, and (3) the right lobe, compromising around 60% of the total liver volume [156]. In some cases, dual grafts (two left lobes from two donors) are transplanted to one recipient [157].

An incision in the right subcostal region is made in all living liver donors, reaching the midline without any dissection of the rectus muscle [158]. Worth mentioning is that the living donor graft is associated with the implantation of a smaller hepatic artery, hepatic vein, and portal vein [159]. 

##### Piggyback Procedure

This procedure is an alternative technique for liver transplantation that aims to avoid venous bypass, in which the recipient’s retro-hepatic IVC can be left in situ, followed by the anastomoses of the donor’s IVC [160].

##### Post-Operative Care

The management of patients following liver transplantation is a major step in the success of the surgery [161]. Management strategy focuses on several points, such as the cardiovascular stability and fluid status, which is mandatory in the first few hours [162]. Despite the fact that hemorrhage is rare following liver transplantation, postoperative hypovolemia may be caused due to hypoalbuminemia, especially in cases of massive ascites [163].

Another focus of post-operative management is towards acute kidney injury, which has a high prevalence following LT [164]. The presence of HRS, hemodynamic instability, renal congestion during the surgery, abdominal hypertension, and immunosuppressive therapy toxication increases the chances of post-operative AKI, with a possibility of a gastrointestinal bleed [165].

Furthermore, the prevention of graft rejection is a major task following LT, despite that rejection is lower than in other solid organ transplantations [166]. Steroids, antimetabolites such as Mycophénolate mofétil and calcineurin inhibitors are the routinely used drugs perioperatively, with adjusted doses based on the hepatic and renal functions, in addition to the level of immunity [167].

Another important step in post-transplant care is the induction of antibiotic and antifungal prophylaxis, in adjustment with the patient’s immune status and the surgical events [168]. Worth mentioning is that pneumonia is a major cause of morbidity following LT, and a cornerstone is deciding the specific antibiotic regimens [169]. Well nutrition is also an important point to consider, to reduce the morbidity post-operation [170].

#### 3.3.4. Long-Term Outcomes and Complications of Liver Transplantation

Complications of LT are classified as either early or late, depending on the timing of occurrence.

##### Early Complications

In the first week, the abnormal liver enzymes usually trend down towards normal, and the liver graft starts to regenerate [171]. However, several complications could disrupt the regeneration process, mostly being the following:

i. Primary non-function of the liver allograft is an immediate complication presenting with the lack of bile production or clear bile production, and it necessitates a new graft following abnormal liver enzymes and bilirubin levels [172].
ii. Hepatic artery thrombosis is an early complication that can also develop later, with a clinical presentation that varies from asymptomatic to fever and increased liver enzymes. It can result in hepatic ischemia, necrosis, and ischemic cholangiopathy, leading to the need for re-transplant [173].
iii. Acute cell rejection occurs in up to 50% of patients in the first 2 months, where most patients respond to corticosteroids, leading to favorable outcomes on the long-term [174].
iv. Biliary complications, mainly biliary strictures, are managed with endoscopic dilation, stenting, or surgical revision [175].
v. Infections, mainly due to the use of immunosuppressive agents, are usually due to opportunistic organisms such as CMV, *Candida* infections, *Pneumocystis carinii*, *Aspergillus*, *Nocardia*, and *Cryptococcus* [168].

##### Late Complications

i. Complications related to immunosuppression, where the most common are chronic kidney disease (CKD), hypertension (HTN), diabetes mellitus (DM), and dyslipidemia [176]. Also, immunosuppressive drugs increase the risk of cardiovascular disease. Corticosteroids may also cause osteoporosis, in the long term, with the additive effect of liver disease on vitamin D [177]. Calcineurin inhibitors may lead to neurologic impairments, such as tremors and paresthesias [178].
ii. Recurrent disease post-liver transplantation, including recurrent infections (hepatitis C or B) and chronic liver diseases (NASH, PBC, PSC, AIH, and HCC) [179].
iii. De novo malignancy, a major cause of death in the long run, is mainly due to several risk factors, including the use of immunosuppression, infections, and alcohol intake [180].

##### Long-Term Outcomes

LT offers an increase in life expectancy in cirrhotic patients. Above 50% of those who had liver transplantation survived for 7 years, compared to 25% in those who died on the waiting list. With advancements, the one-year survival rates increased from its start till 2014 when they reached 90%, after which the improvement reached a plateau. The median survival time had an 8-year increase following cadaveric LT [181]. A study performed on two centers in Nottingham and Ottawa found that the 1-, 5-, 10- and 20-year survival rates were 98%, 95%, 87%, and 62%, and 100%, 96%, 88%, and 62%, respectively [182]. However, the success rate is not homogeneous between centers with high and low cases. A study performed in Korea found that centers with more than 50 liver transplantations/year had better outcomes than centers with less than 50 cases per year, such as in-hospital and long-term mortality rates [183]. LT assures a good quality of life in liver recipients, as long as they maintain high therapeutic adherence following the transplantation process [184].

### 3.4. Prognosis and Quality of Life Considerations

#### 3.4.1. Survival Rates and Factors Influencing Prognosis

The estimated rate for ten-year survival rate in patients with compensated cirrhosis is 47% and with decompensated cirrhosis is 16%. As for the one-year survival rate, the classification based on Child–Turcotte–Pugh (CTP) scoring, the one-year survival rates were 100% for group A, 80% for group B, and 45% for group C. As for the survival following liver transplantation, the one-year and five-year rates were around 85% and 72%, respectively [185].

The prognosis of liver cirrhosis was affected by several factors, most importantly the presence of complications. After the occurrence of complications, the analysis of mortality prognosis found that gender, the etiology of cirrhosis, the CTP stage, marital status, the primary caregiver, and follow-up management affect the prognosis [186].

#### 3.4.2. Strategies to Improve Patient Quality of Life

Liver cirrhosis is associated with poor quality of life with no significant difference between the various etiologies. The presence of complications and their severity also play an important role in shaping the quality of life of cirrhotic patients, in addition to the effect of the disease-specific symptoms that are normally present, such as pruritus, muscle cramps, sleep disturbance, fatigue, and gastrointestinal symptoms [7]. Advanced stages of chronic liver disease, old age, female sex, low socioeconomic status and financial burden were found to lower the quality of life, while good health perception was found to have a better one, regardless of the stages of liver disease [187]. Liver transplantation is the major enhancer for quality of life, as it is the only definitive treatment for advanced cases [188]. Other types of therapies, despite improving the survival and reducing the mortality rate, had only minor effects, lacking any significant role on the quality of life [7].

## 4. Conclusions

Cirrhosis is associated with several complications with a variable level of severity, ranging from mild to life-threating. These complications are accompanied by a high rate of mortality and a low quality of life. The best management strategy for such complications starts with preventive measures, mainly through dietary monitoring and lifestyle modification. Early diagnosis is also of great value, where screening procedures should be performed routinely due to the availability of management approaches for most of the complications at early stages. However, complications in advanced stages require liver transplantation. Despite the advancements in surgical techniques aiming to maximize the use of organs and to supply the biggest number possible of patients on the waiting list, to our date, the need for organs highly exceeds the number available. This highlights the need for early detection and intervention to minimize the number of patients in need. Recent scientific findings and research advancements led to the improvement of therapeutic and diagnostic approaches, yet more accurate screening options are still lacking, in addition to definitive therapeutic strategies for some complications.

## Figures and Tables

**Table 1:** Prevalence, screening methods, and therapeutic approaches for complications of liver cirrhosis.

Complication	Prevalence in Decompensated Cirrhosis	Screening	Treatment
Ascites [78,79]	50%	Clinical diagnosis	Moderate ascites: Diuresis + mineralocorticoids + salt restriction Large ascites: Paracentesis + albumin infusion
Esophageal varices [80,81]	85%	Esophagogastroduodenoscopy	Prophylaxis: Nonselective beta blocker Treatment: Endoscopic band ligation
Hepatic encephalopathy [82]	40%	Clinical diagnosis	Reverse precipitant nutritional support medications: Lactulose for treatment and prophylaxis, rifaximin (Xifaxan) added for prophylaxis
Hepatocellular carcinoma [83]	No data	Ultrasound every six months	LT, systemic treatments (mainly immunotherapy)
Hepatorenal syndrome [84,85]	20%	Clinical diagnosis	Splanchnic vasoconstrictors Terlipressin (or Octreotide) + Albumin
Bacterial peritonitis [86]	30%	Clinical diagnosis, Paracentesis, Ascites fluid neutrophil count	Antibiotics for treatment and prophylaxis
Malnutrition [87]	50%	Clinical diagnosis	Multivitamin correct diet
Abdominal hernia [88]	20%	Clinical diagnosis	Surgery
Leg cramps [89]	67%	Clinical diagnosis	Manage electrolytes with baclofen

**Table 2:** Doses of diuretics used for the treatment of ascites in cirrhotic patients.

Diuretic	Initial Dose	Maximal Dose
Spironolactone	100 mg daily	400 mg daily
Furosemide	40 mg daily	160 mg daily
Amiloride	15–30 mg daily	-

**Table 3:** The use of antibiotics for empiric treatment of SBP.

Antibiotic	Use
3rd generation cephalosporin (cefotaxime or ceftriaxone)	Community-acquired SBP
Piperacillin–tazobactam	Community-acquired SBP or Healthcare or nosocomial SBP (areas of minimal multidrug resistance)
Meropenem with glycopeptides (or daptomycin)	Healthcare or nosocomial SBP (areas of high multidrug resistance)
Ceftolozane–tazobactam and ceftazidime–avibactam	Carbapenem-resistant species Extended spectrum beta-lactamase producing Gram-negative bacteria Multidrug-resistant *Pseudomonas aeruginosa* Multidrug-resistant *Acinetobacter* spp.

**Table 4:** The different vasoconstrictors used for managing hepatorenal syndrome in cirrhotic patients.

Vasoconstrictor	Type	Effect
Terlipressin and octreotide	Splanchnic vasoconstrictor	Reduce portal inflow Improve filling of the central compartment
Midodrine and norepinephrine	Systemic vasoconstrictor	Improve renal circulation

## Data Availability

No new data were created or analyzed in this study. Data sharing is not applicable to this article.
